# Preoperative flexion-extension radiographs can't exclude c1/c2 reducibility in c1/c2 subluxation

**Published:** 2012-11

**Authors:** Mohammad Samadian, Reza Jabbari, Shahab Khazanehdari

**Affiliations:** ^*a*^Loghman-Hakim Hospital, Shahid Beheshti University of Medical Sciences, Tehran, Iran.

## Abstract

**Background:**

According to the findings of previous studies, treatment of C1/C2 subluxation especially in congenital anomaly depends on preoperative dynamic radiograph and site (anterior or posterior) of cord compression. Evolution of new technique for instrumentation intraoperative manipulation is more feasible. We present our experience of treating 5 cases “apparently” irreducible atlantoaxial subluxation by intraoperative reduction by “C2 Spinus Process Compression Maneuver”and restoration of craniovertebral region alignments.

**Methods:**

During Sep 2004 to Nov 2012, a total of 8 cases with C1 C2 subluxation Due to Os odontoidum (4 cases) and Basilar invagination (2 cases), trauma (2 cases) involving the craniovertebral junction were treated in our department of neurosurgery. Two patients had basilar invagination and apparently 'fixed' atlantoaxial dislocation and four patients had osodontoidum and reducible atlantoaxial dislocation. The patients ranged from26 to 54 years in age. Neck pain and spastic quadriparesis were the most prominent symptoms. During surgery before instrumentation at least 50% of reduction was achieved by “C2 Spinus Process Compression Maneuver”, then C1 - C2 or Occ_C1 - C2 instrumentation and fusion.

**Results:**

Following surgery all the patients showed symptomatic improvement and restoration of craniovertebral alignments. Follow- up ranged from 7 to 39 months (average 26 months).

**Conclusions:**

We recommend posterior surgical approach for reduction and instrumentation for C1/C2 subluxation even when anterior compression is more than posterior compression. Intraoperative “C2 Spinous Process Compression Maneuver” can predict the reducibility of subluxation.

**Keywords:**

C1/C2, Subluxation, Reduction, Dynamic Radiograph


					Volume 4, Suppl. 1 Nov 2012
Publisher: Kermanshah University of Medical Sciences
URL: http://www.jivresearch.org
Copyright:
Congress of Iranian Neurosurgeons, 14-16 November 2012, Kermanshah, Iran
Paper No. 75
Preoperative flexion-extension radiographs can't exclude
c1/c2 reducibility in c1/c2 subluxation
Mohammad Samadian a,*
, Reza Jabbari a
, Shahab Khazanehdari a
a
Loghman-Hakim Hospital, Shahid Beheshti University of Medical Sciences, Tehran, Iran.
Abstract:
Background: According to the findings of previous studies, treatment of C1/C2 subluxation especially in congenital
anomaly depends on preoperative dynamic radiograph and site (anterior or posterior) of cord compression.
Evolution of new technique for instrumentation intraoperative manipulation is more feasible. We present our
experience of treating 5 cases "apparently" irreducible atlantoaxial subluxation by intraoperative reduction by "C2
Spinus Process Compression Maneuver"and restoration of craniovertebral region alignments.
Methods: During Sep 2004 to Nov 2012, a total of 8 cases with C1 C2 subluxation Due to Os odontoidum (4 cases)
and Basilar invagination (2 cases), trauma (2 cases) involving the craniovertebral junction were treated in our
department of neurosurgery. Two patients had basilar invagination and apparently 'fixed' atlantoaxial dislocation
and four patients had osodontoidum and reducible atlantoaxial dislocation. The patients ranged from26 to 54 years
in age. Neck pain and spastic quadriparesis were the most prominent symptoms. During surgery before
instrumentation at least 50% of reduction was achieved by "C2 Spinus Process Compression Maneuver", then C1 - C2
or Occ_C1 - C2 instrumentation and fusion.
Results: Following surgery all the patients showed symptomatic improvement and restoration of craniovertebral
alignments. Follow- up ranged from 7 to 39 months (average 26 months).
Conclusions: We recommend posterior surgical approach for reduction and instrumentation for C1/C2 subluxation
even when anterior compression is more than posterior compression. Intraoperative "C2 Spinous Process Compression
Maneuver" can predict the reducibility of subluxation.
Keywords:
C1/C2, Subluxation, Reduction, Dynamic Radiograph
* Corresponding Author at:
Mohammad Samadian: Loghman-Hakim Hospital, Shahid Beheshti University of Medical Sciences, Tehran, Iran. Tel: 09123262849,
Email: mdsamadian@gmail.com, (Samadian M.).

				

